# Does non-adherence to DMARDs influence hospital-related healthcare costs for early arthritis in the first year of treatment?

**DOI:** 10.1371/journal.pone.0171070

**Published:** 2017-02-02

**Authors:** Annelieke Pasma, Charlotte Schenk, Reinier Timman, Adriaan van ‘t Spijker, Cathelijne Appels, Willemijn H. van der Laan, Bart van den Bemt, Robert Goekoop, Johanna M. W. Hazes, Jan J. V. Busschbach

**Affiliations:** 1 Department of Rheumatology, Erasmus MC, University Medical Center Rotterdam, Rotterdam, the Netherlands; 2 Department of Psychiatry, section Medical Psychology and Psychotherapy, Erasmus MC, University Medical Center Rotterdam, Rotterdam, the Netherlands; 3 Department of Rheumatology, Amphia hospital, Breda, the Netherlands; 4 Department of Rheumatology, Sint Maartenskliniek, Nijmegen, the Netherlands; 5 Department of Pharmacy, Sint Maartenskliniek, Nijmegen, the Netherlands; 6 Department of Rheumatology, Haga hospital, the Hague, the Netherlands; Brigham and Women's Hospital, UNITED STATES

## Abstract

**Introduction:**

Non-adherence to disease-modifying antirheumatic drugs (DMARDs) is suspected to relate to health care costs. In this study we investigated this relation in the first year of treatment.

**Methods:**

In a multi-center cohort study with a one year follow up, non-adherence was continuously measured using electronic monitored medication jars. Non-adherence was defined as the number of days with a negative difference between expected and observed opening of the container. Cost measurement focused on hospital costs in the first year: consultations, emergency room visits, hospitalization, medical procedures, imaging modalities, medication costs, and laboratory tests. Cost volumes were registered from patient medical files. We applied multivariate regression analyses for the association between non-adherence and costs, and other variables (age, sex, center, baseline disease activity, diagnosis, socioeconomic status, anxiety and depression) and costs.

**Results:**

Of the 275 invited patients, 206 were willing to participate. 74.2% had rheumatoid arthritis, 20.9% had psoriatic arthritis and 4.9% undifferentiated arthritis. 23.7% of the patients were more than 20% non-adherent over the follow-up period. Mean costs are € 2117.25 (SD € 3020.32). Non-adherence was positively related to costs in addition to baseline anxiety.

**Conclusion:**

Non-adherence is associated with health care costs in the first year of treatment for arthritis. This suggests that improving adherence is not only associated with better outcome, but also with savings.

## Introduction

Reviews have shown that in rheumatoid arthritis (RA), 49% to 99% of patients are adherent, depending on the measurement method of adherence [1]. Up till now, it is unclear what the actual impact of non-adherence to disease-modifying antirheumatic drugs (DMARDs) is to direct health care expenditures. Non-adherence to DMARDs is suspected to increase health care costs [2]. The aim of this study is 1) to examine the magnitude of the health care costs for inflammatory arthritis in the first year after diagnosis and 2) to determine whether non-adherence to DMARDs has an impact on health care costs.

Health care expenditures for rheumatoid arthritis care comprise of 0.6% of the Dutch healthcare expenditures [3]. These costs consist of 51% medication and aids for rheumatoid arthritis, 19% elderly care, 18% hospital care and 9% primary care (GP visits) [3]. Healthcare costs for RA impose a burden on individual RA patients, health services and society [4]. Studies suggest that drug treatment reduces overall healthcare costs by reducing patients’ need for expensive medical services such as hospitalization and emergency room (ER) treatment [5]. This observation also suggests that improved adherence reduces health care costs.

Over the last decades the outcome for early arthritis has improved tremendously, since it is treated timely and intensively, following the treat to target principle [6, 7]. The primary target for treatment is to reach a state of clinical remission or at least a state of low disease activity. With a timely and intensive treatment in the first year after diagnosis, remission can be reached. This intensive treatment will benefit the long term disease outcome. Therefore, drug therapy is given in an early phase, which consists of DMARDs and corticosteroids [7]. Until the desired treatment target is reached, drug therapy should be adjusted at least every 3 months [6, 7].

When treatment with conventional, synthetic DMARDs fails, a first step-up will be made to higher DMARD dosages or additional DMARDs. This can, for some patients, lead to undesirable side effects, such as gastro-intestinal problems, liver or kidney abnormalities [8]. When this occurs, patients may be referred to other medical specialists, which causes more health care expenditures. When step-ups to conventional DMARDs fail, a step-up to treatment with advanced, but also much more expensive biologicals will be made. That suggests that especially in the first year of treatment, adherence is related to treatment success and costs.

Non-adherence can be expected to cause either more or less health care costs. Usually, the relation in which non-adherence leads to ineffective treatment and higher costs due to substituting expensive treatment, is emphasized. Indeed, the burden of a complex and inconvenient dosing regimen, which commonly causes side-effects, has a negative impact on adherence to treatment and this can hamper to achieve the full benefits of the therapy and logically to poorer long-term outcomes [9–12]. Symptoms and complications may worsen, leading to increased use of hospital and emergency room (ER) services, office visits, and other medical resources [13]. Non-adherence can also imply that money has been wasted for unused medication [10].

On the other hand, non-adherence to DMARDs might also lead to less experienced side-effects. Patients reported that if side-effects outweigh the experienced benefits of the treatment, this is one of the reasons for them to stop taking the medication [14]. This might mean that non-adherent patients are less often referred to medical specialists because of adverse events. From previous studies it is also known that a small amount of patients are not only non-adherent to medication, but also to rheumatologist appointments. These patients avoid health care consumption and might therefore cause even less direct health care expenditures, regardless of possible worsening of their disease activity.

In this study we investigated the hospital costs of PsA, RA and undifferentiated arthritis in the first year after diagnosis and its association with adherence.

## Patients and methods

### Patients

**Fig 1:**
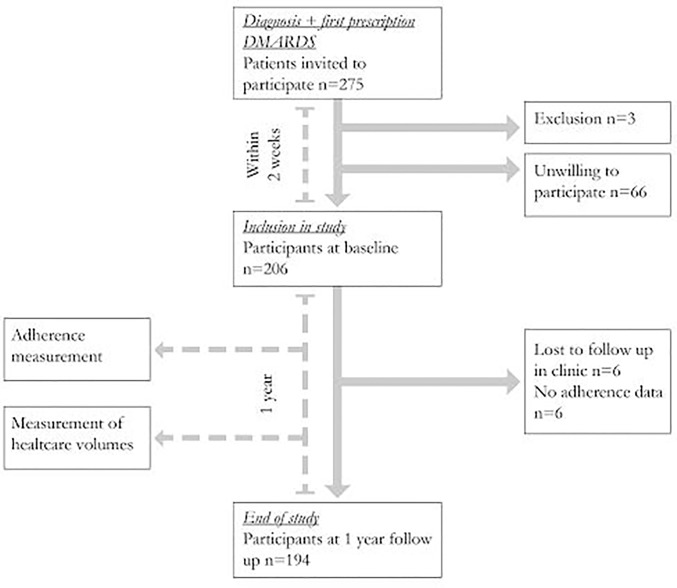
Flowchart of respondents.

From an ongoing adherence cohort study with a one year follow-up, we selected the patients who had finished their participation in the study between March 2013 and December 2014. Patients were recruited in 11 regional hospitals in the Southwest of the Netherlands. The hospitals consisted of one academic hospital, one specialized clinic and 9 general hospitals. Patients were included if they were newly diagnosed with RA, psoriatric arthritis (PsA) or undifferentiated arthritis, started using DMARD therapy for the first time, were at least 18 years old, and were able to read and understand sufficient Dutch. Fig 1 shows the timeline and study set-up. Within two weeks of starting DMARD therapy, patients were included in the study. Clinical variables were assessed at baseline (diagnosis, symptom duration before diagnosis, anti-cyclic citrullinated peptide antibodies (ACPA), Rheumatoid factor and joint involvement) and every three months (28-joint count disease activity score (DAS28)) by a specialized rheumatology nurse or a research nurse. At baseline, patients filled out the Hospital Anxiety and Depression scale (HADS) [15], which consists of two subscales: one for anxiety and one for depression. The scores range between 0 and 21, higher scores indicating more symptoms of anxiety or depression. At baseline, the Health Assessment Questionnaire (HAQ) [16] was filled out to measure physical functioning. This self-administered questionnaire is a validated measure of disability which includes 20 specific functions that are grouped into categories: dressing and grooming, arising, eating, walking, personal hygiene, reaching, gripping and other activities. The average of these scores represents a physical functioning score. HAQ scores range from 0 (no difficulty) to 3 (unable to do).

### Non-adherence measurement

Non-adherence was continuously measured using Medication Event Monitoring System (MEMS) devices, which consist of a medication vial and a MEMS lid with a microprocessor to record the day and time of each vial opening. Participants in the study received at the start of the study for each DMARD a MEMS, which was filled by the pharmacist or the specialized nurse.The data stored in the MEMS lid was every three months transferred by the specialized nurse into a web-based data platform. This data platform compiles hour-by-hour drug dosing histories over extended periods, also noting medication regimen changes. Nursing and medical staff were blind to the adherence data throughout the study.

Extra openings of the MEMS cap will be ignored, because these are mostly not representing medication intake, but openings by pharmacists. This could otherwise lead to an overestimation of adherence. Each day when the medication cap was not opened when it should have been opened, was assigned as a non-adherence event. When a patient stopped taking their DMARD medication on rheumatologist advice, for example in the case of lab abnormalities, this was not assigned as a non-adherence event. For the whole one-year period an underuse proportion was calculated by adding all days in which non-adherence occurred and dividing these by the number of days in the observation period. If a patient used multiple DMARDs in the one-year follow-up period, the mean of the DMARD underuse proportions was calculated.

### Ethics statement

The Erasmus MC Medical Ethics board approved this study. The hospitals’ board of directors of the Bronovo, Haga hospital, Groene Hart, Amphia, Sint Maartenskliniek, Sint Antonius, Reinier de Graaf Gasthuis, Sint Franciscus Gasthuis, Lievensberg and Franciscus hospital gave their consent for participation in the study. All participants gave written informed consent for their participation and for looking up clinical data in their patient files.

### Estimating direct healthcare volumes

In health economics, preferably all costs associated with the treatment are included. This would include not only the treatment costs made in the hospital, but also medical costs made outside the hospital, travel costs and costs of productivity loss. However, in this investigation we had only access to hospital files. These hospital files contain information about care at the department of rheumatology and the other departments in the hospital.

Healthcare volumes were extracted from the patient hospital files by two investigators (AP, CVS) from the date of diagnosis until one year after diagnosis. We only extracted information from the patient files from the hospital in which the patient went to the rheumatology outpatient clinic. The number and type of DMARDs (including prednisone) used were derived from an online system in which the rheumatology nurse had entered the prescribed DMARDs, dosage and regimen during the one year follow up period.

To gain more insight in which costs are affected by adherence, the healthcare volumes were divided into three categories: rheumatology outpatient clinic care, rheumatology referral hospital care (including rheumatology outpatient clinic care) and total hospital healthcare volumes (including the rheumatology clinic and rheumatology referral care). Healthcare volumes were subdivided into: a) consultations (including telephonic patient consultations), b) medical procedures (therapeutic as well as diagnostic), c) imaging modalities, d) admissions (including day admissions), and e) ER visits. For rheumatology outpatient healthcare, we also subdivided into f) laboratory costs, and g) medication costs (costs for synthetic and biologic DMARDs, costs for prednisone). For visits to other specialists, no data was available on laboratory costs and medication costs.

The number of comorbidities per patient was measured as the number of separate medical specialists the patient went to without being referred by the rheumatologist. Due to time constraints it was impossible to register per individual patient all types of blood tests that were conducted in one year. Because the standard strategies of rheumatology lab monitoring differ per hospital, we randomly selected a number of 10 patients per hospital to determine which set of laboratory tests are commonly conducted. We calculated the total costs for these test sets and then counted per patient how many times laboratory tests were requested for monitoring.

### Unit prices

To assign unit prices to the different cost categories we used costs derived from the Dutch manual for cost of illness studies [17] and the Dutch price list for medical treatments, supplement 2 [18]. For medication costs, we used the Dutch price list for medication [19]. In case of the existence of different medicine manufacturers, the mean medication price was used. All unit prices were corrected for inflation to June 2014 using the inflation numbers from the Central Bureau of Statistics [20].

### Statistical analyses

We used univariate descriptive measures to report demographic and disease characteristics of the study population. Statistical comparison of the baseline characteristics between patients lost to follow up and patients with complete follow up were made with Student t-tests and chi square tests. Cost data is usually skewed, with some patients making much more costs that the mode. To allow for a multivariable analysis, we transformed the cost and adherence data, since both are heavily skewed. A rank transformation was chosen to allow for normal distribution of the residuals. Multivariable linear regressions were conducted with the three cost categories as dependent variables and non-adherence (rank tranformed), baseline anxiety, baseline depression, number of comorbidities, education level, baseline disease activity as measured with the DAS28, age, gender and diagnosis as possible predictors. Predictors other than non-adherence were chosen based on previous research (e.g. anxiety and depression are well known predictors of healthcare costs). Furthermore, age and the number of comorbidities, as well as baseline disease activity were chosen as possible predictors since they are expected to affect healthcare costs. Current standard care guidelines differ per diagnosis and thus may indirectly affect healthcare costs and is therefore a covariate. Because of the explorative character of the analysis, we choose to enter all covariates in the multivariable regression at once. For the association between non-adherence and costs, x-rays of the hand and feet were not included, because they were taken for most patients, but sometimes just within the one-year timeframe and sometimes just outside the timeframe. This could otherwise lead to an over- or underestimation of the associations between variables and costs. They were, however taken into account in the description of the costs.

To visualize the association between non-adherence and costs, non-adherence was categorized per 0.05 non-adherence proportion, resulting in an ordinal scale with 20 categories. The mean patient costs were plotted per non-adherence category.

Non-adherence was also dichotomized using an 80% adherence cut-off. The proportional distribution of costs were visualized in pie charts for adherent and non-adherent patients. Mann-Whitney U tests were used to compare the median costs per category for adherent and non-adherent patients.

### Missing data

Patients were excluded from analysis when a patient became lost to follow up in the clinic, and the hospital files did not include the healthcare consumption of the whole year. For some patients, adherence data was incomplete because of lost to follow up in the study. If a patient had less than one month of adherence data, the patient was excluded from analysis. For patients who had less than one year monitoring data, the mean underuse proportion for the observed amount of days was used in the analysis and was thus held to be constant for the remainder of the study period. We also did a complete case analysis including only patients with more than 11 months of adherence observations to check the robustness of our findings. The disease activity from patients who were lost to follow up from the study was extracted from the patient files.

A p-value below 0.05 was considered as statistical significant, and all analyses were performed using SPSS, version 21.0 (IBM Corp. released 2012. IBM SPSS Statistics for Windows, Version 21.0. Armonk, NY: USA).

## Results

### Patients

Of the 275 invited patients, 206 were willing to participate. Twelve patients were lost to follow up either in the clinic or during the study period and were excluded from analysis, which left 194 patients with complete cost data (Fig 1). Of the 206 patients who were included in the study, for 158 (81.4%) patients, 1-year adherence data was available, for 173 patients, more than 200 days of adherence monitoring data was available.

In Table 1, the demographic and disease characteristics of the study population are presented. Most patients (74.2%) had rheumatoid arthritis. There were no statistical significant differences between those patients lost to follow up and patients that completed the cohort, except for the mean HADS anxiety score, which is much higher for the patients who became lost to follow up than for the patients with complete follow up (10.0 versus 5.4; p = 0.015).

**Table 1:** Demographic and disease characteristics and adherence percentages.

	Total (n = 206)	Patients with 1-year follow up (n = 194)	Patients lost to follow up (n = 12)
Age in years, mean (SD)	53,7 (14.2)	54 (14)	46.3 (16.4)
Gender, female, n (%)	130 (63.1)	123 (63.4)	7 (58.3)
Type of hospital, n (%)			
• General • Academic	175 (84.9) 31 (15.1)	165 (85.1) 29 (14.9)	10 (83.3) 2 (16.7)
Diagnosis, n (%)			
• RA • PSA/ arthritis with Crohn • other	153 (74.2) 43 (20.9) 10 (4.9)	145 (75.9) 41 (21.1) 8 (4.1)	8 (66.7) 2 (16.7) 2 (16.7)
Baseline DAS28, mean(SD)	4.24 (1.36)	4,26 (1,36)	3.87 (1.43)
Baseline HAQ, median (IQR)	0.75 (0.29–1.13)	0.75 (0.25–1.13)	0.94 (0.69–1.34)*
Education level, n (%)			
• Low • Medium • High	87 (42.2) 63 (30.6) 43 (20.9)	85 (43.8) 61 (31.4) 41 (21.1)	2 (33.3)* 2 (33.3)* 2 (33.3)*
HADS anxiety, mean SD	5.6 (4.5)	5.4 (4.4)	10 (5.3)*
HADS depression, mean SD	4.5 (3)	4.5 (3)	5.6 (3.4)*
Medication characteristics			
Subcutanuous MTX, n (%)	39 (18.9)	37 (19.1)	2 (16.7)
Use of biologicals, n (%)	20 (9.7)	19 (9.8)	1 (8.3)
Mean 1-year non-adherence proportion (1 = non-adherent)			
• MTX • PRED • SSZ • HCQ • ARA	0.3 (n = 194)	0.14 (n = 184)	# (n = 10)
	0.17 (n = 70)	0.12 (n = 65)	# (n = 5)
	0.22 (n = 31)	0.17 (n = 28)	# (n = 3)
	0.19 (n = 47)	0.15 (n = 45)	# (n = 2)
	0.03 (n = 2)	0.03 (n = 2)	-

Abbreviations: DAS28: 28 joint count Disease Activity Score; HAQ: Health Assessment Questionnaire; DMARDs: Disease-modifying Anti-Rheumatic Drugs; MTX: methotrexate, PRED: prednisone; SSZ: sulfasalazine; HCQ: hydroxychloroquine; ARA: arava

#: no adherence data was available

*6 patients had missing data

### Health care costs

The average costs for the rheumatology outpatient clinic over the one-year period are € 1455.76 (SD €2402.04), the average one-year costs including referrals are € 1620.47 (SD € 2471.14) and the average total costs for this patient group are approximately € 2117.25 (SD € 3020.32) per year. As expected, among the patients, there was high variability in health care consumption, which results in skewed data. The number of patients using the various types of health care is given in Table 2. The mean number of rheumatology visits is 4.64 (range 2–11) and the mean number of specialized rheumatology nurse visits is 3.2 (range 0–5). Of the imaging modalities, x-rays were mostly used in the rheumatology outpatient clinic (83.7%), followed by ultrasound (10.5%). Therapeutic procedures in the rheumatology outpatient clinic only consist of intra-articular or intra-muscular corticosteroid injections.

**Table 2:** Components of healthcare costs for early arthritis.

	Rheumatology outpatient clinic	Mean costs	Rheumatology referrals	Mean costs	Comorbidities	Mean costs
***Consultations with medical specialist***						
No. of patients	196 (100%)		74 (37.8%)		62 (31.6%)	
Mean no. per patient ± sd	4.6 ± 1.8	€ 245.87	3.2 ± 3.4	€ 295.24	4.3 ± 4.6	€ 401.09
***Consultations with specialized nurse/nurse practitioner***						
Mean no. per patient ± sd	3 ± 2.51	€ 100.70	N/A		N/A	
***Imaging modalities***						
No. of patients	84 (42.9%)		17 (8.8%)		34 (17.5%)	
Mean no. per patient ± sd	3.1 ± 2.0	€ 198.94	2.12 ± 1.5	€ 295.86	2.12 ± 1.95	€ 254.28
***Medical procedures***						
*Diagnostic procedures*						
No. of patients	11 (5.7%)		40 (20.6%)		27 (13.9%)	
Mean no. per patient ± sd	1.18 ± 0.4	€ 122.59	1.8 ± 1.3	€ 70.65	1.59 ± 1.0	€ 162.67
*Therapeutic procedures*						
No. of patients	52 (26.8%)		2 (1%)		3 (1.5%)	
Mean no. per patient ± sd	1.44 ± 0.7	€ 8.54	15 ± 7.0	#	2 ± 1	##
***ER visits***						
No. of patients	2 (1%)		3(1.5%)		16 (8.2%)	
Mean no. per patient ± sd	1 ± 0	€ 163.75	1 ± 0	€ 163.75	1.31 ± 0.6	€ 214.92
***Hospital admissions (including day admissions)***						
No. of patients	9 (4.6%)	€ 6082.38	4 (2.1%)	€ 607.84	22 (11.3%)	€ 2488.24
***Laboratory tests***						
Mean no. per patient ± sd	8 ± 4.6	€ 146.93	N/A		N/A	

Abbreviations: SD: standard deviation, N/A: not applicable, ER: emergency room

# no unit price available

## Not all the unit prices were available (2 out of 3 not available)

Referrals to other specialists by rheumatologists were given to 75 patients (38.7%), ranging from 1 to 4 different specialists. Most referrals were to dermatology (16), pulmonary specialists (12), eye care specialists (12) and orthopedic surgeons (14). Diagnostic procedures in the category ‘rheumatology referrals’ were mostly for tuberculosis screening for patients who needed a step up in therapy to biological use.

Sixty-three patients (32.5%) went to other medical specialists for comorbidities, ranging from 1 to 8 different specialists per patient. Most occurring specialist visits for comorbidities were surgery (14), cardiology (10), eye care (8), dermatology (8), and internal medicine (8).

### Non-adherence

Adherence differed per DMARD type. Using an 80% adherence cut-off, for MTX, 77.7% of patients were adherent, as for hydroxychloroquine 77.8% were adherent. For prednisone, 80.0% of patients were adherent and for sulfasalazine, 71.4% were adherent.

Most patients started treatment with a combination of 2 DMARDs. During the first year of treatment, 20 patients (10.2%) were switched to biologic DMARDs and 40 patients (20.4%) were switched from oral to subcutaneous use of MTX.

**Fig 2:**
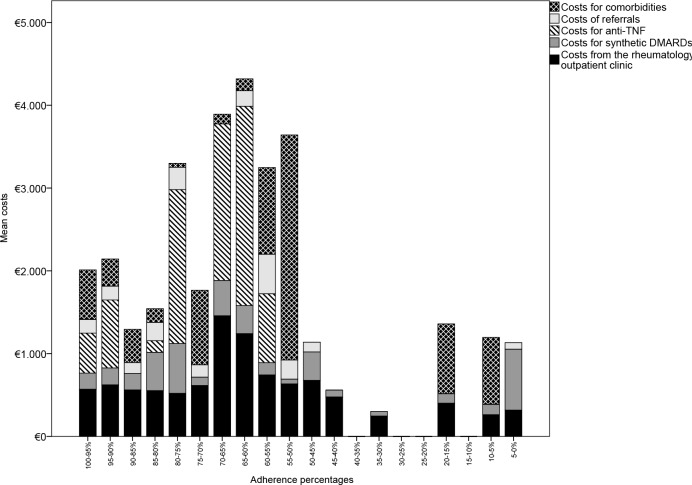
Association between costs and adherence percentage.

Fig 2 depicts that as the adherence percentage decreases from 100% to 60% (40% of the amount of medication not taken), the mean costs increase as well. However, this relation disappears when patients are more than 40% non-adherent. Note that the patients who are more than 40% non-adherent are a small minority; the overall study population is adherent: 90.7% of the patients are between 100 and 60% adherent. More than 75% of the study population is more than 80% adherent. The increase in costs with the increase of non-adherence seems to be driven by the costs of anti-TNF. This is probably because some non-adherent patients start using anti-TNF early, and these drugs are substantially higher priced.

**Fig 3:**
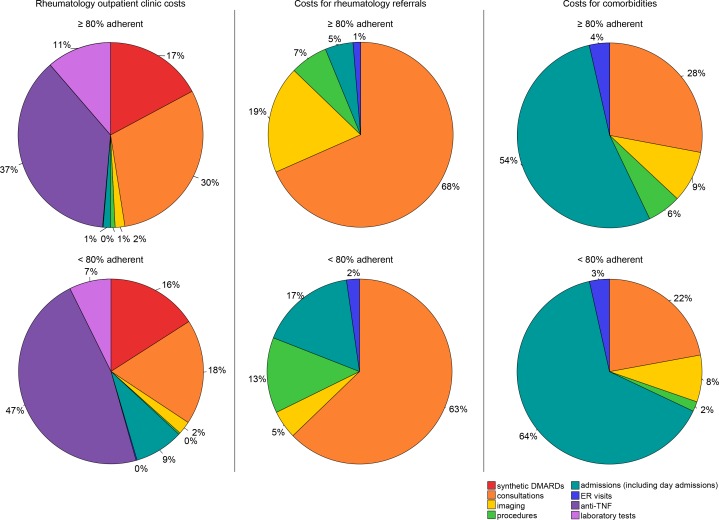
Percentage distribution of costs categories for patients 80% or more adherent and patients less than 80% adherent.

Fig 3 depicts the distribution of the costs for adherent and non-adherent patients. An adherence cut-off point of 80% is used. Patients who are less than 80% adherent make more costs for anti-TNF. In all three cost categories, patients who are less than 80% adherent have relatively more costs for hospital admissions than adherent patients. However, the medians of the costs for anti-TNF do not significantly differ.

### Associations with costs

Table 3 shows the results of the multivariate regression of non-adherence on costs. Non-adherence is, corrected for other covariates, statistically significant associated to higher total costs, higher costs made at the rheumatology outpatient clinic, and higher rheumatology-related costs.

**Table 3:** Multivariable linear regression analysis of possible predictors of costs per cost category.

	Rheumatology outpatient clinic costs		Rheumatology-related costs		Total hospital costs	
	Standardized Beta	p-value	Standardized Beta	p-value	Standardized Beta	p-value
Non-adherence	**0.253**	**0.001**	**0.181**	**0.020**	**0.188**	**0.006**
Age	0.146	0.106	0.166	0.069	**0.212**	**0.008**
Gender	0.061	0.430	0.032	0.680	0.045	0.510
Education level	-0.019	0.821	-0.043	0.607	-0.046	0.534
Baseline HADS anxiety	**0.217**	**0.025**	**0.208**	**0.033**	**0.193**	**0.025**
Baseline HADS depression	0.047	0.628	0.035	0.716	0.021	0.808
Baseline DAS28	0.039	0.638	0.122	0.143	0.128	0.082
PsA	0.326	0.061	0.255	0.146	**0.310**	**0.044**
RA	0.300	0.084	0.198	0.257	0.232	0.132
Nr of comorbidities	0.077	0.320	0.025	0.752	**0.408**	**<0.001**

Abbreviations: HADS: Hospital Anxiety and Depression Scale, PsA: psoriatic arthritis, RA: rheumatoid arthritis, DAS28: 28-joint count disease activity score

In a sensitivity analysis, we only included patients with complete adherence data (more than 11 months of observed data) in the regression analyses. Non-adherence remained statistically significant associated to all three cost categories.

## Discussion

This is the first study to find evidence that non-adherence is associated with hospital health care costs in the first year of treatment of arthritis. In addition to non-adherence, baseline symptoms of anxiety are associated to hospital health care costs.

The mean number of visits to the rheumatologist is slightly less than previous studies on health care consumption in a rheumatoid arthritis cohort, that found an average number of 5.7 visits to the rheumatologist per year [4]. However, in this cohort, not the health care consumption in the first year of treatment, but that of established patients was investigated. Health care consumption is expected to be higher in the first year of treatment than in the years thereafter, since treatment has to be tailored and adjusted in the first period of disease, and therefore more visits to the rheumatologist are needed.

The percentage of patients referred to other specialists for arthritis- or DMARD related symptoms was 38.7%. The percentage of patients with comorbidities in our cohort was 32.5%, which is slightly higher than found in other studies (27%) [21], which might explain why our costs in the first years were higher. The difference in comorbidities can be due to the fact that we measured comorbidities as the number of different medical specialists visited instead of the number of additional diagnoses.

Non-adherence is, corrected for other covariates, associated with higher healthcare costs at the rheumatology outpatient clinic, higher rheumatology-related costs, and higher total healthcare costs. From the data that we collected, it does not appear that patients who are non-adherent make more costs in terms of visits to health care specialists or that they are referred more often to healthcare specialists: the relationship between non-adherence and costs found is related to higher medication costs. It seems that patients who were switched to subcutaneous methotrexate or anti-TNF were non-adherent to their oral DMARD medication. It could be that because of non-adherence, their disease activity escalated and that they were switched sooner to more expensive medicines such as anti-TNF.

Although there is not much empirical research about the relation between non-adherence and costs in rheumatology in practice, numerous authors suggest that being non-adherent would lead to higher healthcare cost [5, 10, 13, 22, 23]. We could confirm this suggestion for most patient that are non-adherent, but for patients who are more than 40% non-adherent, costs seem to be lower. These patients do not significantly differ from the more adherent patients in diagnosis, but it might be that these patients have a lower baseline disease activity. Over the course of one year, the disease activity of these patients is slightly lower than the disease activity of the more adherent patients. It might be that being non-adherent is a response to experiencing low disease activity. It might also be that these patients do not have to visit the rheumatologist as often because of mild disease.

Because this is a cross-sectional study, there is uncertainty about the direction of the causality between healthcare costs and non-adherence. It might be that higher healthcare costs lead to non-adherence, but this seems unlikely since in the Netherlands, all rheumatologist care is covered by healthcare insurance. All patients have healthcare insurance and therefore have the same healthcare costs. Confounding is however a likely scenario. It could for instance be that medication side effects result in both high costs and non-adherence.

In addition to non-adherence, there is also a relationship between baseline symptoms of anxiety and healthcare costs. The relationship between healthcare costs and anxiety is well-known [24, 25].

We were not able to include all costs in our analysis. We had only access to hospital files, and have no data on out of pocket costs and costs of productivity loss. Patient with recent onset arthritis are often on sick leave because of high disease activity, which would contribute to productivity losses and thus to higher societal costs [26–28]. Other studies have suggested that non-adherence does decrease work productivity [29, 30]. It could be that if we had access to this data of productivity loss, the association between non-adherence and costs might have been larger. Also, costs for supplemental drugs to prevent NSAID induced symptoms and over the counter medication were not measured. They might also attribute to higher costs in RA [27, 31].

MEMS is up till now the best indirect method to measure non-adherence, and is considered as a ‘gold standard’. Because it measures behavior ‘real time’, it is a very accurate measure. The disadvantage of using MEMS, is that it does not prove ingestion of medication. Participants were instructed to use the MEMS vials for each separate DMARD, but we cannot be sure that they all took their DMARDs from the MEMS vials all the time, which might lead to an overestimation of adherence. Also, we could not measure adherence from patients who were switched to subcutaneous MTX or biologicals, because these medicines do not fit in the medication vial. Subcutaneous MTX is prescribed when the patient experiences too many gastro-intestinal side-effects from oral MTX. Patients who used subcutaneous MTX were asked to put their folic acid in the MEMS vial, so that adherence could still be measured. Furthermore, of the 39 patients who received subcutaneous MTX, 21 also used an additional DMARD to which adherence could still be measured. Biologicals are in the first year of treatment prescribed when the target of treatment was not reached with synthetic DMARDs and there are prognostically unfavorable factors present [6]. Biologicals are mostly added to the treatment with synthetic DMARDs, thus adherence to the synthetic DMARDs could still be measured. Electronic measurement of adherence is sometimes seen as an intervention itself and might increase adherence behavior, but this effect is regarded as small [32].

The outcomes of this study might be subjected to the ‘adherer effect’ [33]. Patients who adhere to the rheumatologists’ prescription have better disease outcomes, regardless of the underlying treatment and are therefore expected to have less health care costs. This theory is based on the finding that behaviors of adherent people are different from the behaviors of non-adherent people. Adherent people have better global health outcomes, since they have more healthy lifestyles, do not engage in risky behaviors and are more adherent to nonpharmacologic prescriptions [34, 35]. Patients who agreed to participate in this cohort study are probably more adherent than the general patient population, which is also known from other studies [14]. This means that in daily practice the effect of non-adherence on costs might be larger.

In addition, patients who became lost to follow up were or became probably less adherent than the patients who completed follow up. The patients in this cohort are rather adherent to their medication and there is little variation in adherence. This makes it more difficult to study the association between non-adherence and hospital costs.

This study shows that there is an association between non-adherence and costs. This suggests that improving adherence is associated with savings. Most money can be saved in medication costs. The mean medication costs for patients who are switched to anti-TNF therapy, are almost 30 times more than the costs for patients who use synthetic DMARDs.

Our findings address the need to improve adherence, because money is being wasted and potentially beneficial medication is discarded. It is important to study which patients are at risk for non-adherence, so that interventions to improve adherence can be targeted. While there remains uncertainty about which patients are at risk and how to intervene on adherence behavior, rheumatologists should at least be aware that patients might be non-adherent to therapy. Focusing on the way they communicate with the patient is important, because the patient-doctor relationship is an inescapable factor in establishing good adherence behavior [36]. The rheumatologist should build up towards a trustworthy relationship with the patient so that communication about non-adherence can take place and the importance of adherence to the treatment can be addressed. This is not only better for the patient, but will also save money from a societal perspective.

## References

[1] Pasma A, van 't Spijker A, Hazes JMW, Busschbach JJV, Luime JJ. Factors associated with adherence to pharmaceutical treatment for rheumatoid arthritis patients: A systematic review. Semin Arthritis Rheum. 2013;43(1):18–28. 10.1016/j.semarthrit.2012.12.001 23352247

[2] Van Den Bemt BJF, Zwikker HE, Van Den Ende CHM. Medication adherence in patients with rheumatoid arthritis: A critical appraisal of the existing literature. Expert Rev Clin Immunol. 2012;8(4):337–51. 10.1586/eci.12.23 22607180

[3] Poos M, Bijenhof A, Slobbe L. Reumatoïde artritis (RA): Hoeveel zorg gebruiken patiënten en wat zijn de kosten?. Volksgezondheid Toekomst Verkenning, Nationaal Kompas Volksgezondheid, versie 4.17 2014 23 june 2014 [cited; Available from: http://www.nationaalkompas.nl/gezondheid-en-ziekte/ziekten-en-aandoeningen/bewegingsstelsel-en-bindweefsel/reumatoide-artritis-ra/zorgkosten/

[4] Verstappen SMM, Verkleij H, Bijlsma JWJ, Buskens E, Kruize AA, Heurkens AHM, et al Determinants of direct costs in Dutch rheumatoid arthritis patients. Ann Rheum Dis. 2004;63(7):817–24. 10.1136/ard.2003.014340 15194577 PMC1755054

[5] Sokol MC, McGuigan KA, Verbrugge RR, Epstein RS. Impact of medication adherence on hospitalization risk and healthcare cost. Med Care. 2005;43(6):521–30. 15908846 10.1097/01.mlr.0000163641.86870.af

[6] Smolen JS, Landewé R, Breedveld FC, Buch M, Burmester G, Dougados M, et al EULAR recommendations for the management of rheumatoid arthritis with synthetic and biological disease-modifying antirheumatic drugs: 2013 update. Ann Rheum Dis. 2014;73(3):492–509. 10.1136/annrheumdis-2013-204573 24161836 PMC3933074

[7] Smolen J, Aletaha D, Bijlsma JWJ, Breedveld FC, Boumpas D, Burmester G, et al Treating rheumatoid arthritis to target: recommendations of an international task force. Ann Rheum Dis. 2010;69(4):631–7. 10.1136/ard.2009.123919 20215140 PMC3015099

[8] Ruderman EM. Overview of safety of non-biologic and biologic DMARDs. Rheumatology (United Kingdom). 2012;51(SUPPL. 6):vi37–vi43. 10.1093/rheumatology/kes283 23221586

[9] Robin Dimatteo M, Giordani PJ, Lepper HS, Croghan TW. Patient adherence and medical treatment outcomes A meta-analysis. Med Care. 2002;40(9):794–811. 10.1097/01.MLR.0000024612.61915.2D 12218770

[10] Golay A. Pharmacoeconomic aspects of poor adherence: Can better adherence reduce healthcare costs? J Med Econ. 2011;14(5):594–608. 10.3111/13696998.2011.597808 21732903

[11] DiMatteo MR. Variations in patients' adherence to medical recommendations: A quantitative review of 50 years of research. Med Care. 2004;42(3):200–9. 15076819 10.1097/01.mlr.0000114908.90348.f9

[12] DiMatteo MR. Evidence-based strategies to foster adherence and improve patient outcomes. JAAPA. 2004;17(11):18–21. 15575518

[13] Kane S, Shaya F. Medication non-adherence is associated with increased medical health care costs. Dig Dis Sci. 2008;53(4):1020–4. 10.1007/s10620-007-9968-0 17934828

[14] Pasma A, Van't Spijker A, Luime JJ, Walter MJM, Busschbach JJV, Hazes JMW. Facilitators and barriers to adherence in the initiation phase of disease-modifying antirheumatic drug (DMARD) use in patients with arthritis who recently started their first DMARD treatment. J Rheum. 2015;42(3):379–85. 10.3899/jrheum.140693 25512473

[15] Spinhoven P, Ormel J, Sloekers PPA, Kempen GIJM, Speckens AEM, van Hemert AM. A validation study of the Hospital Anxiety and Depression Scale (HADS) in different groups of Dutch subjects. Psychol Med. 1997;27:363–70. 9089829 10.1017/s0033291796004382

[16] Boers M, Jacobs JWG, van Vliet Vlietland TPM, van Riel PLCM. Consensus Dutch Health Assessment Questionnaire. Ann Rheum Dis. 2007;66:132–3. 10.1136/ard.2006.059451 17178759 PMC1798401

[17] Hakkaart-van Roijen L, Tan SS, Bouwmans CAM. Handleiding voor kostenonderzoek, methoden en standaard kostprijzen voor economische evaluaties in de gezondheidszorg: College voor zorgverzekeringen; Geactualiseerde versie 2010.

[18] Tarieflijst Instellingen 2012, supplement 2. In: Zorgautoriteit N, ed. Utrecht, the Netherlands: Nederlandse Zorgautoriteit 2011.

[19] Medicijnkosten. 2014 1 october 2014 [cited 2014 22 october]; Available from: http://www.medicijnkosten.nl

[20] Mjaavatten MD, Radner H, Yoshida K, Shadick NA, Frits ML, Iannaccone CK, et al Inconsistent treatment with disease-modifying antirheumatic drugs: A longitudinal data analysis. J Rheum. 2014;41(12):2370–8. 10.3899/jrheum.140306 25320216 PMC4843119

[21] Kroot EJJA Van Gestel AM, Swinkels HL Albers MMC, Van de Putte LBA, Van Riel PLCM. Chronic comorbidity in patients with early rheumatoid arthritis: A descriptive study. J Rheum. 2001;28(7):1511–7. 11469455

[22] Osterberg L, Blaschke T. Adherence to medication. NEJM. 2005;353(5):487–97. 10.1056/NEJMra050100 16079372

[23] Iuga AO, McGuire MJ. Adherence and health care costs. Risk Manag Healthc Policy. 2014;7:35–44. 10.2147/RMHP.S19801 24591853 PMC3934668

[24] Boulanger L, Zhao Y, Bao Y, Russell MW. A retrospective study on the impact of comorbid depression or anxiety on healthcare resource use and costs among diabetic neuropathy patients. BMC Health Serv Res. 2009;9. 10.1186/1472-6963-9-111 PMC2719623 19566952

[25] Vasiliadis HM, Dionne PA, Préville M, Gentil L, Berbiche D, Latimer E. The excess healthcare costs associated with depression and anxiety in elderly living in the community. Am J Geriatr Psychiatry. 2013;21(6):536–48. 10.1016/j.jagp.2012.12.016 23567409

[26] Doeglas D, Suurmeijer T, Krol B, Sanderman R, Van Leeuwen M, Van Rijswijk M. Work disability in early rheumatoid arthritis. Ann Rheum Dis. 1995;54(6):455–60. 7632086 10.1136/ard.54.6.455 PMC1009902

[27] Van Jaarsveld CHM, Jacobs JWG, Schrijvers AJP, Heurkens AHM, Haanen HCM, Bijlsma JWJ. Direct cost of rheumatoid arthritis during the first six years: A cost-of-illness study. Brit J Rheumatol. 1998;37(8):837–47. 9734674 10.1093/rheumatology/37.8.837

[28] Geuskens GA, Hazes JMW, Barendregt PJ, Burdorf A. Work and sick leave among patients with early inflammatory joint conditions. Arthritis Care Res. 2008;59(10):1458–66. 10.1002/art.24104 18821663

[29] Hovinga CA, Asato MR, Manjunath R, Wheless JW, Phelps SJ, Sheth RD, et al Association of non-adherence to antiepileptic drugs and seizures, quality of life, and productivity: Survey of patients with epilepsy and physicians. Epilepsy Behav. 2008;13(2):316–22. 10.1016/j.yebeh.2008.03.009 18472303

[30] Wagner S, Lau H, Frech-Tamas F, Gupta S. Impact of medication adherence on work productivity in hypertension. Am J Pharm Benefits. 2012;4(4):e88–e96.

[31] Jacobs J, Keyserling JA, Britton M, Morgan GJ Jr, Wilkenfeld J, Christina Hutchings H. The total cost of care and the use of pharmaceuticals in the management of rheumatoid arthritis: The medi-cal program. J Clin Epidemiol. 1988;41(3):215–23. 3123614 10.1016/0895-4356(88)90124-2

[32] Sutton S, Kinmonth AL, Hardeman W, Hughes D, Boase S, Prevost AT, et al Does Electronic Monitoring Influence Adherence to Medication? Randomized Controlled Trial of Measurement Reactivity. Ann Behav Med. 2014:1–7. 10.1007/s12160-014-9595-x PMC4223537 24573909

[33] Simpson SH, Eurich DT, Majumdar SR, Padwal RS, Tsuyuki RT, Varney J, et al A meta-analysis of the association between adherence to drug therapy and mortality. BMJ. 2006;333(7557):15–8. 10.1136/bmj.38875.675486.55 16790458 PMC1488752

[34] Canner PL, Forman SA, Prud'homme GJ. Influence of adherence to treatment and response of cholesterol on mortality in the Coronary Drug Project. NEJM. 1980;303(18):1038–41. 10.1056/NEJM198010303031804 6999345

[35] Dormuth CR, Patrick AR, Shrank WH, Wright JM, Glynn RJ, Sutherland J, et al Statin adherence and risk of accidents a cautionary tale. Circulation. 2009;119(15):2051–7. 10.1161/CIRCULATIONAHA.108.824151 19349320 PMC2744446

[36] Haskard Zolnierek KB, Dimatteo MR. Physician communication and patient adherence to treatment: A meta-analysis. Med Care. 2009;47(8):826–34. 10.1097/MLR.0b013e31819a5acc 19584762 PMC2728700

